# A Pleiotropic Role of the Hepatitis B Virus Core Protein in Hepatocarcinogenesis

**DOI:** 10.3390/ijms222413651

**Published:** 2021-12-20

**Authors:** Caroline Lefeuvre, Hélène Le Guillou-Guillemette, Alexandra Ducancelle

**Affiliations:** 1 Laboratoire de Virologie, Département de Biologie des Agents Infectieux, CHU Angers, F-49000 Angers, France; HeLeguillou@chu-angers.fr (H.L.G.-G.); AlDucancelle@chu-angers.fr (A.D.); 2 HIFIH Laboratory UPRES EA3859, SFR ICAT 4208, Angers University, F-49000 Angers, France

**Keywords:** hepatitis B virus, hepatitis B virus core protein, hepatocellular carcinoma, hepatocarcinogenesis

## Abstract

Chronic hepatitis B virus (HBV) infection is one of the most common factors associated with hepatocellular carcinoma (HCC), which is the sixth most prevalent cancer among all cancers worldwide. However, the pathogenesis of HBV-mediated hepatocarcinogenesis is unclear. Evidence currently available suggests that the HBV core protein (HBc) plays a potential role in the development of HCC, such as the HBV X protein. The core protein, which is the structural component of the viral nucleocapsid, contributes to almost every stage of the HBV life cycle and occupies diverse roles in HBV replication and pathogenesis. Recent studies have shown that HBc was able to disrupt various pathways involved in liver carcinogenesis: the signaling pathways implicated in migration and proliferation of hepatoma cells, apoptosis pathways, and cell metabolic pathways inducing the development of HCC; and the immune system, through the expression and production of proinflammatory cytokines. In addition, HBc can modulate normal functions of hepatocytes through disrupting human host gene expression by binding to promoter regions. This HBV protein also promotes HCC metastasis through epigenetic alterations, such as micro-RNA. This review focuses on the molecular pathogenesis of the HBc protein in HBV-induced HCC.

## 1. Introduction

Hepatitis B virus (HBV) infection is a major health problem worldwide despite the availability of an effective vaccine and antiviral drugs. More than 295 million individuals are chronic HBV carriers, and are at risk of developing end-stage liver diseases (cirrhosis, liver failure, and hepatocellular carcinoma [HCC]) [1,2,3,4].

HBV is an enveloped virus belonging to the Hepadnaviridae family with a 3.2 kb circular and partly double-stranded DNA genome [5]. The transcriptional template of HBV is the covalently closed circular double-stranded DNA (cccDNA), which resides inside the hepatocyte nucleus as a chromosome-like structure. The cccDNA, which is its viral persistence form, expresses at least six overlapping RNAs, from four overlapping open reading frames (ORFs), leading to the production of seven proteins. The replication of HBV implicates reverse transcription of the pregenomic RNA (pgRNA) intermediate into HBV DNA. The four ORFs are called C, S, P, and X. The C ORF has two genes that encode the hepatitis B core protein (HBc) and the hepatitis B e (HBe) protein. The S ORF contains preS1, preS2, and hepatitis B surface (HBs) domains, and encodes three viral envelope proteins, including large, middle, and small HBs antigen (HBsAg), respectively. The P and X ORFs have viral polymerase and HBx genes responsible for expressing the polymerase protein and the viral transactivator HBx protein, respectively. The HBV gene expression is regulated by four promoters and two enhancer elements. Enhancer I partially overlaps the X promoter, and enhancer II is located upstream of the core promoter [6,7].

HCC is the sixth most prevalent cancer among all cancers worldwide and ranks the second in annual cancer mortality rates [8]. Chronic HBV infection is the dominant global cause of HCC accounting for 55% of HCC cases worldwide and 80% or more of these in the Eastern Pacific region and Sub-Saharan Africa, which are the areas with the highest tumor incidence [9,10].

Previous studies have suggested that chronic HBV infection results in the integration of the HBV genome into the host chromosome, persistent liver inflammation, high levels of reactive oxygen species, continual hepatocyte regeneration, and the dysregulation of the cell death and DNA repair pathways in the liver, which may in turn contribute to the development of HCC through hepatocyte destruction/regeneration and malignant transformation [11,12,13]. Hepatoma formation is linked to significant changes in several cellular signaling pathways, including the Wnt/β-catenin, p53, MAPK, and NF-kB pathways, and it alters the expression of these genes [14].

**Figure 1:**
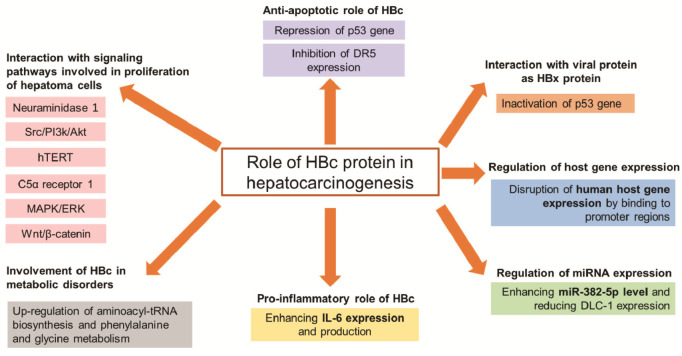
Pleiotropic role of HBc protein in hepatocarcinogenesis. DR5: death receptor 5; NEU1: neuraminidase 1; hTERT: human telomerase reverse transcriptase; C5AR1: C5α receptor 1; MAPK/ERK: mitogen-activated protein kinase/extracellular signal-regulated kinase; IL-6: interleukine-6; HBV: hepatitis B virus; HCC: hepatocellular carcinoma; DLC-1: deleted in liver cancer.

To date, the molecular mechanisms of HBV malignant transformation remain to be comprehensively elucidated. The pathogenesis of the tumor seems to be multifactorial, and the mechanisms involved include activation of oncogenes and silencing of tumor suppressor genes. Among specific proteins of HBV, much of the available evidence supports the hypothesis that the HBx protein plays a pathogenetic role for the mechanisms underlying HBV-induced malignant transformation [8]. HBx is a key regulatory protein of HBV that modulates transcription, protein degradation, signal transduction, and apoptotic and cell cycle regulatory pathways [15]. Here, we are interested in the HBc protein, another protein of HBV that contributes in nearly every stage of the HBV life cycle due to the versatile nature of this protein, in particular through its C-terminal arginine-rich domain and the post-translational modifications occurring at this level [16]. HBc protein could also be involved in the malignant transformation process. Thus, HBc is found to be associated with the activation of cellular signaling pathways that are related to cell proliferation and migration [14,17,18,19]. Some studies also suggest the role of HBc protein arising from its gene regulatory properties [20] and HBc proteins have been found expressed in enormous quantities in infected tissues [14]. The principal purpose of this review is to illustrate and understand the complex role of HBc protein in HBV malignant transformation. While the previous reviews focused on the role of HBx protein in the molecular mechanisms of HBV malignant transformation, this review, for the first time, focuses on the involvement of HBc protein in the development of HCC (Figure 1 and Table 1).

## 2. HBc Protein, Description, and Functions in the Viral Life Cycle

HBc protein, also named core antigen, is a structural component of the viral nucleocapsid. The 21 kDa HBc protein self-assembles to form the subviral nucleocapsid particles that package the viral polymerase and pgRNA during RNA replication [27,28]. HBc assembles first in dimers, secondly in trimers of dimers and finally into hexamers forming icosahedral capsid particles [29]. Multimerization of 120 HBc dimers (i.e., 240 capsomeres) leads to the production of the icosahedral capsid particle. The dimers form the icosahedral capsid with a triangulation number T = 3 for 90 self-assembling dimers, or T = 4 for 120 self-assembling dimers [30].

Depending on the HBV genotype (A to J), HBc is a 183 or 185 residue protein, with two distinct domains connected by a hinge region (position 140 to position 149): the N-terminal 140 amino acid domain contains the capsid assembly domain that is sufficient for self-assembly into capsid particles; and the C-terminal arginine-rich domain (CTD) (position 149 to position 183 or 185), which shares a high similarity with protamine and functions as a nucleic acid-binding domain (RNA/DNA viral) [31,32]. The core protein has been found in both the cytoplasmic and nuclear compartments of HBV-infected hepatocytes according to histological analysis of tissues from HBV-infected patients [33,34]. Posttranslational modifications are important in targeting specific subcellular compartments, regulating the folding of proteins, their stability, their interaction with ligands or other proteins, and their catalytic activity or signaling function [35]. These modifications include C-terminal serine phosphorylation, ubiquitination, and arginine methylation in particular [36]. Thus, the HBc CTD plays a potential role in HBc subcellular localization. The nuclear import of capsids is facilitated by HBc phosphorylation [16]. The HBc CTD undergoes dynamic phosphorylation and dephosphorylation events that regulate its nucleic acid binding, subcellular localization, and other functions, such as pgRNA packaging, DNA synthesis, and virion secretion [16,37]. Arginine methylation is involved in a variety of biological processes including chromatin regulation, transcription control, RNA processing, and nuclear transport [36]. Therefore, the core protein contributes in nearly every stage of the HBV life cycle, including subcellular trafficking, and release of the HBV genome, RNA metabolism, capsid assembly, and transport, and regulation of viral reverse transcription [16]. Moreover, several studies have suggested that HBc might function as a gene regulatory protein based on the presence of nucleic acid-binding motifs, nuclear localization signals, phosphorylation sites at the serine residues of the C-terminal SPRRR motif and its localization in the nucleus [38,39,40]. HBc could regulate various biological processes by interacting with different cellular factors [41], such as human gene promoters, and could disrupt normal host gene expression as a regulator of transcription [20].

## 3. HBc Protein, a Pleiotropic Role in Hepatocarcinogenesis

### 3.1. Interaction with Signaling Pathways Involved in Proliferation of Hepatoma Cells

The development of HBV-mediated HCC involves deregulation in many cellular signal pathways and the identification of these pathways is important in understanding the pathophysiological role of proliferation and migration of hepatoma cells in HCC [42].

#### 3.1.1. Neuraminidase 1 Pathway

Neuraminidase 1 (NEU1) has been described as a major target in the sialidase-mediated regulation of tumorigenesis. This human sialidase expressed in a variety of tissues regulates the sialylation of multiple proteins. It participates in many physiological processes, including cellular proliferation, migration, differentiation, and apoptosis [43,44]. NEU1 expression is elevated in HCC tissues [45] and some studies found that this sialidase is associated with HCC induced by HBV infection [46,47].

One study aimed to evaluate the role of NEU1 in the activation of signaling pathways and epithelial–mesenchymal transition, and the proliferation and migration of hepatoma cells mediated by HBc protein. They showed via immunohistochemical analysis that NEU1 was upregulated in HBV-positive hepatoma cells and HBV-related HCC tissues because HBV promotes NEU1 expression via HBc protein in hepatoma cells. Through the increase in NEU1 expression, HBc contributes to the activation of downstream signaling pathways and epithelial–mesenchymal transition in HBV-associated hepatoma cells. NEU1 therefore facilitates the proliferation and migration of hepatoma cells mediated by HBc protein, which may in turn upregulate NEU1 to facilitate HCC development. These data provide novel insights into the molecular mechanism underlying the hepatocarcinogenesis mediated by HBc and indicate that NEU1 plays an important role in HBc-mediated functional abnormality in HCC [18]. The involvement of NEU1 with oncogenic viruses other than HBV has not been described so far.

#### 3.1.2. Abnormal Sarcoma (Src)/PI3k/Akt Pathway

Aberrant phosphoinositide 3-kinase (PI3k)/Akt pathway activation would be associated with the development of HCC [48]. As a dominant non-receptor tyrosine kinase activated in HCC, abnormal sarcoma (Src) signaling activation conferred by HBV is involved in HBV-mediated HCC [49,50]. The HBV large surface antigen (LHBs) and the HBV core protein, two proteins of HBV, seem to activate this pathway. LHBs promotes liver carcinogenesis by activating the Src/PI3K/Akt pathway. These effects were caused by activation of this pathway through proximal stimulation of PKCα/Raf1 signaling by LHBs.

Following these findings, a recent study with mechanistic investigations demonstrated that the activation of the Src/PI3k/Akt pathway through proximal switch from inactive Src to the active form of the kinase by HBc protein increased the tumor formation of hepatoma cells. HBc-mediated Src kinase activation was associated with downregulation of C-terminal Src kinase (Csk). In addition, HBc enhances Src expression by activating the alternative Src 1A promoter in a Sp1 transcription factor-dependent manner. In other words, the HBV core protein serves to promote Src kinase activation by repressing the expression of Csk at a transcriptional level and increasing Src expression by facilitating the Sp1 transcription factor. Proliferation induced by stable HBc expression is associated with increased G1-S cell cycle progression mediated by Src kinase activation. These findings reveal that the HBV core protein is a strong prosurvival factor and serves to promote tumorigenesis of hepatoma cells dependent on Src/PI3k/Akt signal activation in vitro and in vivo [14].

#### 3.1.3. Human Telomerase Reverse Transcriptase (hTERT) Pathway

The telomerase activation is essential for cell immortalization and the enzymatic activity is regulated by human telomerase reverse transcriptase (hTERT) [51,52]. Aberrant telomerase activity is closely associated with the development of human cancers and senescence evasion [53]. As for other oncogenic viruses, telomerase activity is essential for Epstein–Barr virus (EBV)-induced cell immortalization to overcome cell senescence and maintain replicative potential [54]. Telomerase activity is especially elevated in the development of HBV-related HCC because high levels of telomerase activity are observed in more than 80% of human HCC samples and nearly 100% of HBV-positive HCC tissues [55,56,57].

hTERT expression is regulated by several transcription factors, including c-Myc, Sp1, E2F, and the Ets family [58]. Previous studies indicated in particular that the hTERT expression and its promoter activity are dependent on the transcription factor of c-Ets2 expression [59,60] and that the c-Ets2 is a critical regulator of hTERT expression [59,61]. High levels of c-Ets2 expression would be associated with the development of HCC [62].

Recent research employing luciferase assays showed that the HBc enhances the hTERT promoter activity in a dose-dependent manner. Further mapping of the hTERT promoter region indicated that the sequence of between −197 and −130 bp in the hTERT promoter contains the potential binding sites for transcription factors of c-Ets2 and was important for the HBc-enhanced hTERT promoter activity. HBc protein contributes to hepatoma cell proliferation by upregulating the c-Ets2-dependent expression of hTERT, associated with higher levels of hTERT and nuclear c-Ets2 expression in HBc-positive HCC samples [17].

#### 3.1.4. C5α Receptor 1 Pathway

C5α receptor 1 (C5AR1) is a main component of complement systems. After binding to complement C5α, the activation of C5AR1 has multiple effects on cell activity, such as the regulation of the differentiation and function of various target cells, and the participation in multiple innate immune responses [63,64]. Several studies have demonstrated that C5AR1 is closely associated with the pathogenesis of a variety of human tumors [65,66,67]. Hu et al. found that C5AR1 expression was significantly elevated in HCC tissues, and it could promote the invasion of hepatoma cells via epithelial–mesenchymal transition mediated by extracellular signal-regulated kinase (ERK)1/2 [68].

In a recent study, Kong et al. described that HBV could promote C5AR1 expression through viral HBc protein in hepatoma cells, and the upregulation of C5AR1 mediated by HBc mainly relied on the NF-κB pathway. Based on the C5AR1, HBc facilitates the activation of intracellular signal pathways (such as c-Jun N-terminal kinase (JNK) and ERK pathways) as well as the expression and secretion of interleukin (IL)-6 in hepatoma cells. The activation of JNK and ERK pathways induced by HBc might affect multiple biological processes to facilitate the development of HBV-related HCC. Therefore, C5AR1 has a major role in the growth and migration of hepatoma cells mediated by HBc [19].

#### 3.1.5. Mitogen-Activated Protein Kinase (MAPK)/ERK and Wnt/β-Catenin Pathways

The mitogen-activated protein kinase (MAPK)/ ERK signaling pathway plays a key role in regulating cell biological functions, such as proliferation, differentiation, and cell survival [69]. The Wnt/β-catenin pathway is also an important signaling pathway in the process of growth and development. Abnormal activation of these two pathways is heavily involved in hepatocarcinogenesis [70]. Thus, dysregulation of these pathways leads to inappropriate cellular behavior and participate in cellular transformation and carcinogenesis [69,71]. The frequency of mutations in the components of the MAPK/ERK signaling pathway is low, but frequent activation of the signaling has been found in HCC patients [71]. The aberrant activation may result from somatic mutations in the genes of the Wnt/β-catenin pathway and/or dysregulation of the Wnt/β-catenin pathway [72].

A study showed that HBc protein could bind to 64 gene promoters of the MAPK pathways and 41 gene promoters of the Wnt/β-catenin signaling pathways, thus participating in the progression of HCC. The HBc binding to many gene promoters may have profound effects on host cellular functions, potentially increasing a cell’s susceptibility to harmful factors, such as carcinogens [20].

### 3.2. HBc Protein, an Anti-Apoptotic Viral Protein

Resistance of HBV-infected hepatocytes to apoptosis is considered one of the major causes in the progression of chronic hepatitis to cirrhosis and ultimately to HCC [23,73]. Apoptosis of HBV-infected hepatocytes is mainly mediated by signaling belonging to the tumor necrosis factor (TNF) protein family, including TNF-α, Fas ligand (FasL), and TNF-related apoptosis-inducing ligand (TRAIL) [74,75]. TNF-α and FasL are considered as death receptor ligands.

#### 3.2.1. Repression of the Proapoptotic p53

FasL induces apoptosis of hepatocytes in both normally functioning liver and in various forms of liver disease [76] and the Fas/FasL system plays an important role in hepatocyte death during HBV infection. In response to DNA damage, the p53 tumor suppressor protein induces either apoptosis or cell cycle arrest at the G1-S. Previous studies reported that Fas transcriptional expression is regulated by p53 protein in hepatoma cells, and the cross-talk between the p53 and Fas-FasL pathways in modulating apoptosis is clinically important [77,78]. HCC may progress through the deactivation of the p53 gene [79,80].

A study demonstrated that the expression of HBx protein in infected cells inhibits the induction of apoptosis by direct interaction with the tumor suppressor p53 [81]. A similar mechanism is observed with HBc protein. Liu et al. therefore found that HBc mediated resistance of human hepatoma cells to agonistic anti-Fas antibody-induced apoptosis. They then identified that HBc significantly downregulated the expression of p53, total Fas and membrane-bound Fas at the RNA and protein levels and reduced FasL at the transcriptional level. In contrast, HBc increased the expression of soluble forms of Fas (sFas) by facilitation of Fas alternative splicing. Mechanistically, HBc-mediated Fas alternative mRNA splicing was associated with the upregulation of polypyrimidine tract-binding protein 1 and the downregulation of Fas-activated serine/threonine kinase. HBc may prevent hepatocytes from apoptosis induced by Fas/FasL system by the dual effects of reducing the expression of the proapoptotic form of Fas and enhancing the expression of the antiapoptotic form of the receptor, which may contribute to the survival and persistence of infected hepatocytes toward the development of chronic HBV infection. HBc is a survival factor capable of protecting cells, such as hepatoma cells from anti-Fas antibody-induced apoptosis through the p53-dependent Fas/FasL signaling pathway [21].

The E2F family of transcription factors plays an essential role in mediating cell cycle progression, particularly those involved in G1-S progression [82], and it has been implicated in the regulation of growth inhibition, differentiation, apoptosis, and oncogenic transformation. Previous studies have shown that E2F1 functions as both an oncogene and a tumor suppressor gene [83,84]. A study revealed that HBc protein is a transcriptional repressor of the human p53 gene. Indeed, an electrophoretic mobility shift assay demonstrated that the binding of HBc to E2F1 reduced the DNA-binding ability of E2F1 at the p53 promoter [22].

#### 3.2.2. TNF-Related Apoptosis-Inducing Ligand (TRAIL) Apoptotic Pathway

Unlike TNF and FasL, TRAIL preferentially induces apoptosis of tumor cells and virus-infected cells, but does not induce apoptosis of normal cells [75,85,86]. Following high-risk human papillomavirus (HPV) infection, viral proteins use different strategies to modulate apoptosis. In particular, the E5 protein of HPV can disrupt TRAIL-mediated apoptosis, which suggests that it may prevent apoptosis of cells at early stages of viral infection [87].

The TRAIL was recently reported to be implicated in hepatocyte death during HBV infection. Interestingly, two HBV proteins, HBx and truncated middle hepatitis B surface protein (MHBs(t)), were found to sensitize hepatocytes to TRAIL-induced apoptosis. Other data showed that HBx enhances TRAIL-induced apoptosis through Bax upregulation, whereas MHBs(t) does this through ERK2 activation [75,88].

In contrast, Liu et al. found that HBc protein had an opposite role in TRAIL-induced hepatocyte apoptosis. Indeed, HBc would be a strong inhibitor of TRAIL-induced apoptosis by blocking death receptor 5 (DR5) expression [89] inducing a decrease of TRAIL-induced apoptosis of human hepatoma cells. The DR5 gene promoter has no typical TATA-box, but has two Sp1 sites responsible for the basal transcription activity of the DR5 gene [90]. Transcription factors such as NF-kB and p53 can regulate the DR5 promoter activity. In hepatoma cell lines expressing the core protein, HBc protein induces a significant reduction in DR5 expression that represses the DR5 promoter activity. Consequently, HBc prevents hepatocytes from TRAIL-induced apoptosis through inhibiting DR5 expression [23]. In practice, if the pro-apoptotic proteins, such as HBx, are majority, HBV-infected hepatocytes may die as a consequence, and fulminant hepatitis may develop. In contrast, if the anti-apoptotic viral proteins, such as HBc predominate, the infected hepatocytes may not undergo apoptosis and chronic HBV infection may ensue.

### 3.3. HBc Protein, a Pro-Apoptotic Viral Protein

HBc protein would prevent hepatocyte from FasL-induced apoptosis by altering the membrane and soluble Fas level, while it would also prevent sensitized TNF-α-induced apoptosis by disrupting the interaction between mitogen-activated protein kinase kinase 7 (MKK7) and receptor of activated protein kinase C 1 (RACK1). RACK1 is described as a scaffold protein that facilitates the phosphorylation of MKK7 by its upstream activators.

One study with ectopic expression of HBc in HepG2 cells and primary hepatocyte cultures reported that HBc abolishes the interaction between MKK7 and RACK1 by competing with MKK7 for binding to RACK1, thereby downregulating TNF-induced phosphorylation of MKK7 and the activation of JNK, an important regulator of TNF-α signaling. Specific knockdown of MKK7 increases the sensitivity of hepatocytes to TNF-induced apoptosis, while overexpression of RACK1 counteracts the proapoptotic activity of HBc. The expression of HBc makes hepatocytes susceptible to TNF-induced apoptosis by disrupting the interaction between MKK7 and RACK1 [91] and this finding suggests a direct role of the core protein in driving liver pathogenesis in chronically infected patients.

### 3.4. HBc Protein, a Viral Protein Involved in Metabolic Disorders

The progression of cancer seems to involve major disorders in cell metabolism [92]. Metabolic disorders are shared by both transformed cells and those infected with viruses, suggesting that metabolic reprogramming is an important hallmark of viral oncogenesis. Viruses handle metabolic pathways and associated-signaling cascades to provide sufficient resources for the production of new virions. Among viruses, chronic Hepatitis C virus (HCV) infection is more associated with metabolic alterations than HBV infection. Indeed, patients with chronic HCV often develop secondary metabolic disorders, such as insulin resistance and steatosis [93].

One study reported that the link between metabolic disorders and HCC could be attributed to the effects of HBV infection and in particular the HBV-encoded proteins [94], such as HBx protein [95]. Recently, multi-omics analyses of HBc transfected cells revealed that HBc protein promotes the expression of multiple metabolic enzymes and the secretion of metabolites from hepatoma cells modifying the metabolic characteristics of HCC cells, and contributes to HBV-related metabolic dysregulation through the modulation of glycolysis and amino acid metabolism. For instance, glycolysis and amino acid metabolism are significantly upregulated by HBc. Max-like protein X (MLX) would be an important protein in glycolysis and lipid biosynthesis in tumorigenesis. Besides, MLX might be recruited and enriched by HBc in the nucleus to regulate glycolysis pathways. Moreover, PGK1 is also upregulated by HBc. A recent study highlighted that PGK1 acted as a protein kinase in coordinating glycolysis and the tricarboxylic acid cycle, which is instrumental in cancer metabolism and tumorigenesis [96]. Therefore, Xie et al. concluded that nine pathways were considered closely related to the development of HCC, including aminoacyl-tRNA biosynthesis and phenylalanine and glycine metabolism [24]. 

Dysregulated cholesterol homeostasis is a characteristic of numerous diseases, including liver fibrosis, and even many cancers. A recent study showed that ethanol and HBV together synergistically enhance cholesterol biosynthesis and decrease cholesterol utilization and its uptake in vivo and in vitro. Thus, HBV is involved in the dysregulation of cholesterol homeostasis and increases hepatic cholesterol deposition in alcoholic fatty liver via the hepatitis B core protein [97]. These changes may contribute to the progression of various coexisting diseases.

### 3.5. HBc Protein, a Pro-Inflammatory Viral Protein

Interleukin (IL)-6 is one of the most significant cytokines involved in hepatic inflammation and hepatocarcinogenesis in patients with liver diseases [98,99,100]. Its role has been described in HCV [101] and HBV infections [100]. Higher serum IL-6 level was an independent risk factor for HCC development in female hepatitis C patients. In the case of HBV infection, high serum IL-6 level was also associated with HCC risk [98] and aspartate aminotransferase [102], and considered as a prognostic indicator in HCC [103]. Previous studies described that human hepatoma cells and hepatic cells secrete IL-6 after activating NF-κB pathway and a MyD88-dependent signaling pathway, whose activation is regulated by protein phosphatase type 2 C alpha in the presence of HBx protein [104,105,106]. An intracellular HBcAg expression model (transfected hepatocyte-like cells) showed that the expression of HBc in hepatocytes enhances IL-6 expression and production (checked by qPCR and ELISA, respectively), which was mediated through activating p38 mitogen-activated protein kinase, extracellular signal-related kinase and NF-κB pathways. Cytoplasmic HBcAg seems to be a viral antigen for immune-mediated liver damage, and HBV-infected parenchymal cells may produce proinflammatory cytokines that are involved in pathogenesis of hepatitis B [25].

### 3.6. HBc Protein, a Regulator of miRNA Expression 

Accumulated epigenetic alterations including histone modification, DNA methylation and non-coding RNA (micro RNA or miRNA, lncRNA) were described to have profound significance in HBV-related carcinogenesis [107]. Through its partially complementary sequence to the 3′-UTR of target mRNAs, miRNAs result in gene silencing via translational repression and/or mRNA degradation and are therefore involved in regulating almost all known physiological and pathological processes [108]. miRNAs play a key role in host-virus interactions [109] and their dysregulation is involved in liver fibrosis and a number of human cancers such as HCC. MiR-122 is the most abundant miRNA in the liver, representing 70% of the total miRNA in hepatocytes [110]. MiR-122 is downregulated in patients with HBV-related HCC, while it is upregulated in patients with HBV chronic infection [111].

HBc protein promotes the hepatocarcinogenesis process through the regulation of some miRNAs. The deleted in liver cancer (DLC-1) gene encodes a Rho-GTPase activating protein and is an important negative regulator for cell motility. Previous studies have demonstrated that DLC-1 functioned as a tumor suppressor gene and downregulation or even loss of DLC-1 expression often occurred in HCC [112]. The DLC-1 gene is potentially targeted by several differentially expressed miRNAs in HBc-introduced cells according to the miRNA-target gene network analysis. Thus, miR-382-5p seems to be significantly upregulated in HBc-overexpressing HCC cells. The HBc protein promotes HCC metastasis through enhancing the miR-382-5p level and reducing DLC-1 expression. The miR-382-5p/DLC-1 axis is essential for HBc-promoted HCC metastasis. These data further showed that, similar to HBx, HBc protein might also play multiple roles in different stages of HCC development [26]. A similar mechanism was observed with HCV. Indeed, miRNAs miR-141 and miR-200a are accentuated in HCV-infected human primary hepatocytes and can target DLC-1 mRNA reducing its expression and then induce HCC development [112].

A part of miRNA is closely related to the stage of liver disease. Indeed, studies have shown that the miRNA circulating in serum or plasma could serve as the role of biomarker for the diagnosis and prognosis of HBV-related diseases [113].

### 3.7. HBc Protein, a Regulator of Host Gene Expression 

HBV targets host genes that are involved in cell survival to escape immune surveillance and facilitate malignant transformation. HBc protein may bind specifically to certain human gene promoters, through either its C-terminal functional domain or its N-terminal assembling domain. Previous research has generated the genome-wide profile of HBc in HBV-infected hepatocytes using chromatin immunoprecipitation microarray studies [20]. This study showed that HBc could bind to 64 gene promoters of the MAPK pathways and 41 gene promoters of the Wnt/β-catenin signaling pathways, whereas these two pathways are known to be critically involved in the development of HBV-related hepatocellular carcinoma. Moreover, the authors suggested that HBc tended to target the regulatory regions of genes with molecular function and malignant transformation in the liver cell repertoire. A previous study highlights that the accumulation of slight effects from HBc binding to many gene promoters may produce quite large effects on host cellular functions, possibly increasing a cell’s susceptibility to carcinogens [114].

Therefore, HBc has the ability to bind gene promoters in the human genome to modulate normal functions of liver cells infected with HBV and HBc could disrupt the expression of nearly 3100 human host genes by binding them to promoter regions [20].

### 3.8. Interaction with Viral Protein as HBx Protein

HBc protein seems to be able to interact with HBx, but the impact of this interaction on hepatocarcinogenesis seems to be controversial in some cases. Kwon et al., in their study of cultured HepG2 cells, showed that HBc and HBx proteins could synergistically repress both the promoter activity and the expression of the human p53 tumor suppressor gene [22]. The inactivation of the p53 gene participates in HCC progress and the synergistic action of these two proteins has an impact on malignant transformation.

In contrast, HBc and HBx proteins could reduce the expression of two Id proteins (Id1 and Id3) whereas Id proteins are supposed to be elevated in many tumor types and would correspond with the poor prognosis of HCC patients. Thus, HBc is capable of restraining the BMP/Smad signaling pathway and HBx is able to interact with both Id proteins for facilitating their degradation through proteasome-dependent manners. Therefore, it would be interesting to explore how HBV, one of the pathogenic factors of HCC, influences Id proteins [115].

Then, HBx protein can transactivate the expression of all HBV proteins through these two enhancers and can therefore increase the expression of the HBc in vitro and in vivo by transactivating the C promoter. The regulation of HBx level may be important in HCC development. In cultured human hepatoma cells, Kim et al. demonstrated that the level of HBx protein was significantly reduced by the co-expression of HBc, whereas the level of HBx mRNA was unaffected. The inhibitory effect of HBc is specific to HBx, and it did not affect other HBV proteins. It seems that HBx activates the synthesis of HBc during the early stage of viral replication and that HBc in turn functions as an effective downregulator of HBx. The regulation of the HBx level could involve the activation of the proteasome-mediated degradation of HBx. To date, no direct physical interaction between HBc and HBx has been demonstrated. Nevertheless, mutational analysis indicated that the C-terminal half of HBc is responsible for its inhibitory effect and that HBc protein controls the HBx level via a form of inhibitory feedback mechanism [116]. This study highlights a novel aspect of HBc function in the HBV life cycle and possibly in the development of HCC through control of the HBx level. To our knowledge, the molecular mechanism was not identified.

All of these elements emphasize a close link between HBc and HBx proteins, with a number of potential impacts on liver tumorigenesis.

## 4. Conclusions and Perspectives

Due to the high morbidity and mortality of HCC worldwide, for a number of years, many investigations on HCC carcinogenesis have been conducted that seek to elucidate the molecular mechanisms facilitating the design of better strategies to treat HCC. Evidence supports that the HBc protein has an oncogenic role in HBV-related HCC through several mechanisms, thereby controlling cancer cell proliferation and enabling malignant transformation. These include the signaling pathways involved in migration/proliferation of hepatoma cells; the resistance of cells to apoptosis; cell metabolic disorders; enhancing IL-6 expression and production; epigenetic alterations (miRNA); and other genetic processes.

With regard to garnering new insights into the biological roles of HBc in regulating HBV-related hepatocarcinogenesis, further exploration of the molecular mechanisms related to the dysfunction of hepatoma cells mediated by HBc may help us find novel therapeutic strategies for HBV-related HCC.

## Figures and Tables

**Table 1:** Interactions and reported mechanisms of HBc protein on the various pathways (signaling pathways involved in migration and proliferation of hepatoma cells, apoptosis pathways, metabolic disorders, immune system), epigenetic and genetic events.

Group	Target	Mechanism of HBc to Promote Hepatocarcinogenesis	Reference
Signaling pathways	Neuraminidase 1	Promote NEU1 expression inducing proliferation and migration of hepatoma cells	[18]
	Src/PI3k/Akt	Activate Src/PI3k/Akt pathway inducing tumor formation of hepatoma cells	[14]
	hTERT	Upregulate the c-Ets2-dependent expression of hTERT inducing hepatoma cell proliferation	[17]
	C5α receptor 1	Upregulate C5AR1 via NF-κB pathway to facilitate the growth and migration of hepatoma cells	[19]
	MAPK/ERK and Wnt/β-catenin	Bind to gene promoters of these pathways, thus participating in the progression of HCC	[20]
Anti-apoptosis	p53	Prevent hepatoma cells from anti-Fas antibody-induced apoptosis through the p53-dependent Fas/FasL signaling pathway	[21]
		Repress the p53 gene through the transcription factor E2F1 binding site in the p53 promoter	[22]
	DR5	Prevent hepatocytes from TRAIL-induced apoptosis through inhibiting DR5 expression	[23]
Metabolic disorders	Cell metabolism	Upregulate aminoacyl-tRNA biosynthesis and phenylalanine and glycine metabolism inducing development of HCC	[24]
Immune system	IL-6	Enhance IL-6 expression and production that involved in pathogenesis of HBV	[25]
Epigenetic	miR-382-5p	Promote HCC metastasis through enhancing miR-382-5p level and reducing DLC-1 expression	[26]
Genetic	Promoter regions	Disrupt human host gene expression by binding to promoter regions, which modulate normal functions of liver cells	[20]
Interaction with viral protein as HBx protein	p53	Inactivate the p53 gene, thus participating in HCC progress	[22]

NEU1: neuraminidase 1; hTERT: human telomerase reverse transcriptase; C5AR1: C5α receptor 1; MAPK/ERK: mitogen-activated protein kinase/extracellular signal-regulated kinase; DR5: death receptor 5; MKK7: mitogen-activated protein kinase kinase 7; IL-6: interleukine-6; HBV: hepatitis B virus; HCC: hepatocellular carcinoma; DLC-1: deleted in liver cancer.

## Data Availability

Not applicable.
